# Oxygen–Ozone Therapy of Musculoskeletal Neck Pain: A Review

**DOI:** 10.3390/jpm14030326

**Published:** 2024-03-21

**Authors:** Jiri Jandura, Milan Vajda, Michal Cech, Pavel Ryska

**Affiliations:** 1 Department of Diagnostic Radiology, University Hospital Hradec Kralove, Sokolska 581, 50005 Hradec Kralove, Czech Republic; jiri.jandura@fnhk.cz (J.J.); milan.vajda@fnhk.cz (M.V.); michal.cech@fnhk.cz (M.C.); 2 Department of Diagnostic Radiology, Faculty of Medicine in Hradec Kralove, Charles University, Simkova 870, 50003 Hradec Kralove, Czech Republic

**Keywords:** ozone, neck pain, spine, intervertebral disc, nucleus pulposus, computed tomography

## Abstract

Minimally invasive oxygen–ozone (O_2_-O_3_) therapy utilizing the biochemical effects of O_2_-O_3_ mixture is commonly used in the treatment of musculoskeletal pain. The literature dealing with O_2_-O_3_ therapy of spinal pain focuses mainly on the lumbosacral region. The aim of this review is to evaluate the efficacy of O_2_-O_3_ therapy in musculoskeletal pain in the neck region. The Medline (PubMed), SCOPUS, Web of Science, and Google Scholar databases were searched for clinical studies, using the free text terms: ozone, neck, cervical, spine, pain, disc, hernia, nucleolysis, paravertebral, treatment, and various combinations of them. In total, seven studies (two randomized controlled trials and five observational studies) were found. These studies dealt with the intradiscal or intramuscular paravertebral application of O_2_-O_3_ mixture in patients with myofascial pain syndrome, cervical disc hernias, and chronic neck pain. All these studies proved a significant decrease in neck pain (evaluated by Visual Analog Scale or Numerical Rating Scale), and most of them showed improvement in functional status (measured by Oswestry Disability Index or Neck Disability Index). In addition, other pain assessment scales and function and quality of life measures (DN4 questionnaire, pain pressure threshold, cervical lateral flexion range of motion, Japanese Orthopedic Association scale, 12- and 36-Item Short Form Surveys, modified MacNab criteria, and analgesic drug intake reduction) were used. Changes in these measurements also mostly supported the efficacy of O_2_-O_3_ treatment. No significant complications of the treatment were reported. The available evidence is sparse, but despite this, the O_2_-O_3_ treatment of musculoskeletal neck pain can be considered potentially beneficial and relatively safe.

## 1. Introduction

### 1.1. Musculoskeletal Neck Pain

By definition, neck pain (NP) is pain perceived as arising in a region bordered superiorly by the superior nuchal line, laterally by the lateral margins of the neck, and inferiorly by an imaginary transverse line through the spinous process of first thoracic vertebra [1]. The age-standardized prevalence rate of NP was 27.0 per 1000 population in 2019 [2]. 

NP can be categorized in several ways, such as duration (acute, ˂6 weeks; subacute ≤3 months; chronic >3 months), etiology or structure, severity, and type (mechanical vs. neuropathic) [3]. Globally, musculoskeletal NP ranks among the conditions most responsible for primary care consultations [4]. Musculoskeletal NP belongs to the most frequent complaints of patients with neuro-musculoskeletal disorders, such as cervical spondylosis and radiculopathy, fibromyalgia, and whiplash-associated disorders [5,6]. However, the differential diagnosis of NP is broad and includes more serious conditions such as fractures, spinal cord and nerve injuries, cancer, infections, and inflammation. When evaluating a patient with NP, the physician must be alert for “red flags” (trauma, rheumatoid arthritis, Down syndrome, spondyloarthropathy, constitutional or infectious symptoms, upper motor neuron lesion, age under 20 years or over 50 years, coexisting chest pain, diaphoresis, or shortness of breath) in the history and physical examination that may indicate the need for urgent testing and intervention [3,7].

Most episodes of acute musculoskeletal NP will usually resolve with or without treatment, but nearly 50% of patients will continue to feel some level of pain or frequent occurrences [3]. A number of approaches have been developed for the conservative treatment of NP, such as steroidal and non-steroidal anti-inflammatory drug (NSAID) treatments, skeletal muscle relaxants, physical therapy, manipulative spinal therapies, acupuncture, and mesotherapy [8,9]. European clinical practice guidelines consistently recommend the following evidence-based treatment options for NP: reassurance, advice and education (including to remain active and exercise), manual therapy in combination with other treatments, referral for exercise therapy/programs and a range of oral analgesics and topical medications, plus psychological therapies or multidisciplinary treatment for specific subgroups of patients. However, the strength of these recommendations is weak or moderate. There is weak evidence for the use of painkillers such as paracetamol, NSAIDs (for acute pain only), opioids (for acute pain only), and neuropathic pain medication [10].

Myofascial pain syndrome (MPS) constitutes an important part of chronic musculoskeletal pain, including NP. MPS is associated with the occurrence of trigger points (MTrP) and tender areas in the muscles or connective tissue and, sometimes, with a local twitch response on MTrP palpation [11]. By applying Simons’ criteria, the diagnosis of MPS relies mainly on the clinical history and a careful physical examination by a trained clinician [12]. The prevalence of MPS is unclear due to a lack of well-defined diagnostic criteria [13]. However, the finding of MTrP in patients with musculoskeletal NP pain is frequent. The prevalence of MTrP in patients suffering due to chronic widespread pain ranges from 30 to 93% [14]. In a study by Cerezo-Téllez et al. [15], the prevalence of MPS among patients with chronic non-specific NP was 100%, with most prevalent active MTrP being identified in the trapezius muscles in 93,4% of subjects. A range of non-invasive and invasive methods have been proposed for the treatment of MPS [16]. A review article by Desai et al. [17] summarizes the efficacy of pharmacological and nonpharmacological treatments of MPS. Pharmacotherapy includes the use of the following drugs: NSAIDs (including diclofenac patch), cyclobenzaprine, thiocolchicoside, gabapentin, pregabalin, amitriptyline, cyclo-oxygenase-2 (COX-2) inhibitors, tramadol, tropisetron, opioids, lidocaine patch, tizanidine, clonazepam, duloxetine, sumatriptan, botulinum type A toxin, ketamine, L-tryptophan, and memantine. The nonpharmacological treatment of MPS encompasses the following: MTrP injections (dry needling, short- or long-acting anesthetics, or steroids), manual therapy, ultrasound, hydrocortisone phonophoresis, transcutaneous electric nerve stimulation, electrical twitch obtaining intramuscular stimulation, magnetic stimulation, and laser therapy. Authors conclude that most MPS treatment options demonstrate a limited body of evidence for their use. More specifically, there is evidence that NSAIDs and COX-2 inhibitors alleviate pain; however, more controlled trials are required to fully determine their role in MPS. Tizanidine, benzodiazepines, and tropisetron appear to demonstrate some limited evidence for their use. Topical diclofenac and lidocaine patches may also have limited efficacy. Thiocolchicoside is a promising agent, still with limited evidence. Multiple studies support the use of dry needling and trigger point injections, but sustainability is likely based on using these therapies judiciously and in conjunction with manual therapies, such as myofascial release. Newer therapies of MPS, such as ultrasound and laser therapy, show promising results [17].

In addition to surgeries, there are several minimally invasive methods developed for the treatment of intervertebral disc hernias (HD). Percutaneous techniques such as percutaneous discectomy, laser discectomy, and nucleoplasty have minimized the invasive nature of surgeries and decreased complications, such as postsurgical infection [18].

Oxygen–ozone (O_2_-O_3_) therapy is a method which utilizes the biochemical effects of O_2_-O_3_ mixture; nowadays, it is commonly used in the treatment of musculoskeletal pain, including spinal pain [19]. The literature concerning the O_2_-O_3_ treatment of spinal pain predominantly focuses on the lumbar region. Also, for this reason, the aim of this paper is to provide a review of the literature dealing with the O_2_-O_3_ treatment of musculoskeletal NP. The O_2_-O_3_ mixture can be injected into intervertebral discs or paravertebral muscles (including MTrP). Therefore, the efficacy of O_2_-O_3_ treatment can be benchmarked in comparison with other minimally invasive therapeutic methods for musculoskeletal NP, such as dry needling or lidocaine injections. In addition, it can be assumed that other applications of O_2_-O_3_ treatment for musculoskeletal NP could be investigated in the future.

### 1.2. History of O_2_-O_3_ Therapy

The first identification of ozone as a distinct chemical compound was achieved by Schönbein in 1839. His work showed that following electrolysis, water emanated an odor at the cathode defined as “the odour of electrical matter”, which was later on defined as “ozone”, from the Greek ozein (odorant) [20,21]. In 1845, de la Rive and Marignac proposed that ozone is an allotropic form of oxygen. In the 20th century, Mulliken and Dewar clarified the ozone molecular structure. The use of ozone in clinical practice was introduced in the past century by Payer, Aubourg, and Wolff. During the First World War, Wolff successfully used ozone to treat gangrenous wounds, suppurating bone fractures, inflammations, and abscesses. The first reliable model of a medical ozone generator, that allowed for the production of a variable and stable O_2_-O_3_ mixture, was developed in 1958 by Hänsler. Subsequently, this invention served as the basis for ozone therapy expansion over the last 40 years. Since then, the therapeutic use of ozone in medicine has been extended to a large number of diseases [20].

### 1.3. Ozone as a Therapeutic Agent

Ozone is highly water-soluble inorganic molecule composed of three oxygen molecules with a molecular weight of 48 g/mol. The molecular structure of ozone is inherently unstable due to the nature of its mesomeric states, which tends to make it difficult to obtain high concentrations. Ozone will often experience transient reactions with itself or water molecules [21,22]. Ozone has the potential to react to and oxidize organic compounds and, when present as an air pollutant, can cause harmful effects on the respiratory tract [20,23]. However, in appropriate concentrations, ozone may act as a beneficial drug. This is because most of the medical ozone dose is almost instantly quenched by the potent antioxidant capacity of blood due to a number of hydrophilic and lipophilic compounds and a variety of antioxidant enzymes [24]. Therefore, correct ozone dosage is essential for the positive effect of O_2_-O_3_ treatment. Only under this precondition can ozone initiate favorable biological reactions and also possibly reverse chronic oxidative stress [25].

### 1.4. Minimally Invasive O_2_-O_3_ Therapy of Musculoskeletal Neck Pain

The extradiscal injection of ozone into the paravertebral muscle adjacent to an HD was first proposed by Verga in 1989, and the intradiscal injection of ozone was first reported in the 1990s by Muto and Avella and other Italian interventional neuroradiologists [26].

A reduction in HD volume is one of the therapeutic aims of the intradiscal administration of medical ozone, as disc shrinkage may reduce nerve root compression. Another reason for using medical ozone to treat HD is its analgesic and anti-inflammatory effects [27]. The intradiscal injection of O_2_-O_3_ mixture is carried out by 18- to 22-gauge needle insertion, followed by direct insufflation of the O_2_-O_3_ mixture (3–10 mL; ozone concentration about 30 µg/mL) at the level of the pathologic intersomatic space under computed tomography (CT) or fluoroscopic guidance [25,28].

Nucleus pulposus represents the target site for the intradiscal application of O_2_-O_3_ mixture. Ozone causes the fragmentation of glycosaminoglycans contained in the nucleus pulposus with the subsequent release of water molecules. This leads to a small reduction in the nucleus volume, as well as a significant reduction in intradiscal pressure, resulting in the recoil of the nucleus and restoration of the disc. This process can be induced in contained discs. On the other hand, in uncontained discs, the nucleus is exposed to the immune system, with antibody-mediated inflammatory reactions leading to resorption and remission of the extruded nucleus by phagocytosis and lysis [29]. In epidural space, ozone acts as an anti-inflammatory agent modulating and hastening the switch from M1 to M2 macrophages, converting an inflammatory phase to a reparative phase [30]. Ozone is also involved in the regeneration of myelin sheaths [31]. The paravertebral approach is based on the injection of an O_2_-O_3_ mixture into the paravertebral muscle, usually at the level of the HD [25,32]. Due to the various beneficial biological effects of ozone (e.g., anti-inflammatory, antioxidant, and immunomodulatory), it is hypothesized that oxygen–ozone therapy could affect pain in patients with myofascial syndrome when applied to the MTrP [33]. Moreover, injection of the O_2_-O_3_ mixture could contribute to the inactivation of MTrP by the mechanical influence on muscle fibers [19,34].

## 2. Materials and Methods

### 2.1. Search Strategy

A search for clinical trials of O_2_-O_3_ therapy for musculoskeletal NP was performed. Medline (PubMed), SCOPUS, Web of Science, and Google Scholar databases were searched for full-text English language articles using the following free text terms: ozone, neck, cervical, pain, disc, hernia, nucleolysis, paravertebral, treatment, and various combinations of them. The search ranged from 01/2000 to 01/2024.

### 2.2. Eligibility Criteria

Types of studies: Randomized controlled trials (RCTs) and non-randomized prospective and retrospective observational studies.

Participants: Patients with musculoskeletal NP, with or without associated radicular pain. Age ≥ 18 years. Duration of pain: chronic (>3 months) or acute/subacute (≤3 months). Exclusion criterion: NP of non-musculoskeletal origin (e.g., tumor, inflammatory, or traumatic etiology).

Types of intervention: Minimally invasive O_2_-O_3_ treatment applied intradiscally or intramuscularly to the neck region, with or without imaging guidance. 

Outcome measures: Changes in pain intensity were measured by the means of the Visual Analog Scale (VAS) or Numerical Rating Scale (NRS). Both scales range from 0 to 10 points (0—no pain, 10—maximum imaginable pain). Functional status (disability) due to spinal pain was assessed using the Oswestry Disability Index (ODI) or the Neck Disability Index, a modification of the ODI more specific to cervical spine. The range of ODI and NDI is 0–50 points (from minimal disability to bedridden/exaggerated symptoms).

## 3. Results

**Figure 1:**
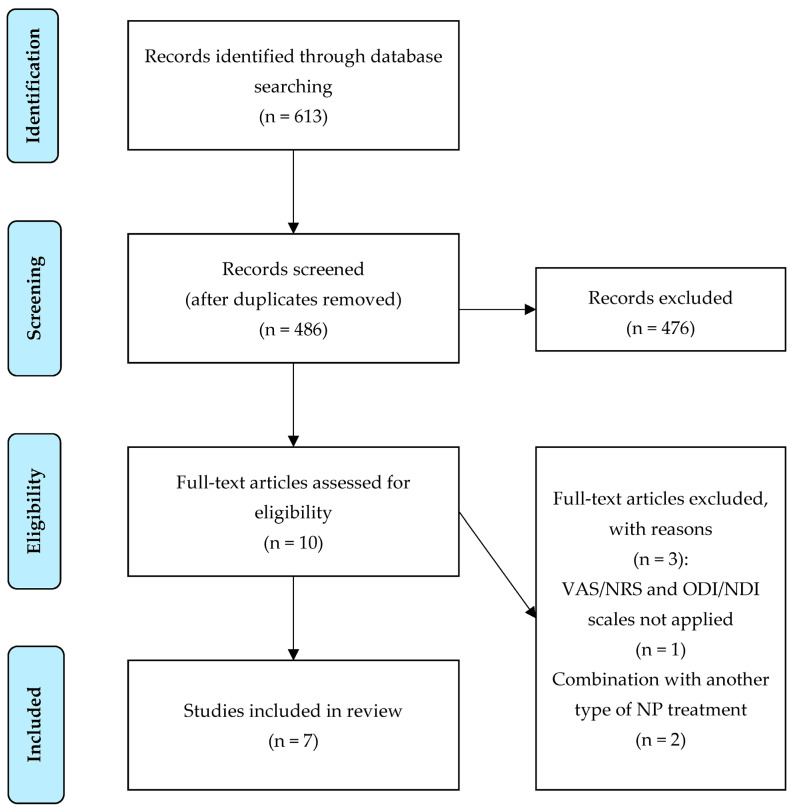
Study selection flowchart. Abbreviations: VAS, Visual Analog Scale; NRS, Numerical Rating Scale; ODI, Oswestry Disability Index; NDI, Neck Disability Index; NP, neck pain.

Two RCTs and five non-randomized observational studies were found. Both RCTs focused on the treatment of neck MPS. Two observational studies (prospective and retrospective) investigated intradiscal O_2_-O_3_ nucleolysis in patients with cervical HD. Three observational studies (one prospective and two retrospective) dealt with the paravertebral intramuscular application of O_2_-O_3_ mixture in patients with NP. The study selection flowchart is marked in Figure 1. A summary of the data from the evaluated studies is shown in Table 1.

### 3.1. O_2_-O_3_ Treatment of Neck MPS

Korkmaz et al. [34] in a single-blind RCT compared the effectiveness of O_2_-O_3_ injection versus lidocaine injection targeted to MTrP in patients with MPS. Both O_2_-O_3_ and lidocaine injections were significantly effective in pain relief and in function improvement measured by VAS, pain score (by applying a pressure on trigger point), and NDI. O_2_-O_3_ ozone injection appeared to be inferior in reducing pain compared to lidocaine injection. No statistically significant change in the cervical lateral flexion range of motion (ROM) was found in both groups.

Raeissadat et al. [13] in a single-blind RCT evaluated the efficacy of O_2_-O_3_ mixture injection applied into the area of MTrP. The effect of O_2_-O_3_ therapy was compared to the injection of lidocaine and dry needling. The authors reported that all three interventions were remarkably effective at improving patients’ pain and the pain pressure threshold (PPT, defined as the minimum amount of compression on the point that reproduces the pain) within 4 weeks after the last injection. However, ROM did not show significant improvements in any group. A statistically significant difference among the evaluated methods was found, favoring O_2_-O_3_ or lidocaine injection in comparison to dry needling. The O_2_-O_3_ group showed a slightly higher improvement in the VAS, PPT, and NDI compared with the lidocaine group, without a statistically significant difference.

### 3.2. O_2_-O_3_ Treatment of Cervical Spine HDs

Ghatge et al. [29] in a prospective non-randomized study investigated the role of ozone disc nucleolysis in cervical HD. All VAS and NDI values were significantly lower at 1-year follow-up compared with pre-treatment levels. The modified McNab criteria for the outcome showed recovery: excellent in 56.1%, good in 20.3%, and fair in 8.9% of patients, resulting in a success rate of 85.4%. MRI performed 6 months after O_2_-O_3_ nucleolysis showed a reduction in HD in 8 of 12 patients compared to pre-treatment MRI.

Beyaz and Sayhan [18] retrospectively evaluated the efficacy and safety of the intradiscal O_2_-O_3_ treatment of cervical HD in patients with chronic NP during a 6-month follow-up. The authors defined the clinical success of O_2_-O_3_ therapy as a 2- and 15-point decrease in VAS and ODI scores compared with baseline values at follow-up terms. Patients reported pain relief at a rate of 93.1%, 95.4%, and 97.7% at 2 weeks, 6 weeks, and 6 months, respectively. Patient satisfaction with treatment was also assessed. At the end of the follow-up, there were 61.3% of patients extremely satisfied, 27.3% fairly satisfied, 9.1% moderately satisfied, and 2.3% poorly satisfied.

### 3.3. Intramuscular Paravertebral O_2_-O_3_ Injection

Rania et al. [35] prospectively evaluated the efficacy and safety of intramuscular paravertebral O_2_-O_3_ injections in patients with cervicobrachial pain. The follow-ups were realized after 8 and 12 sessions of O_2_-O_3_ therapy and one year after treatment finished. Pain intensity was evaluated using NRS. The DN4 and SF-36 surveys were used to assess neuropathic pain and quality of life. The development of adverse drug reactions was also recorded. Statistically significant improvements in quality of life with a decrease in pain were observed in follow-up terms. All patients were pain free after one year. Patients significantly reduced the use of drugs. No serious adverse drug reaction was recorded.

Ucar et al. [36] in a retrospective observational multicentric study evaluated pain scores in patients who underwent paravertebral O_2_-O_3_ injections for NP caused by cervical disc disease. Significant improvements were observed in VAS and Japanese Orthopedic Association scores (JOAs) at both 2 and 6 months in comparison to the pre-treatment scores. There was no significant difference in the VAS or JOAs between 2 and 6 months after O_2_-O_3_ injection.

Latini et al. [32] retrospectively evaluated the effects of O_2_-O_3_ intramuscular paravertebral injections in patients with chronic NP or low back pain. The outcomes in patients with chronic NP were evaluated using NRS, NDI, and SF-12 survey. Significant beneficial effects of O_2_-O_3_ therapy were observed during the 6-month follow-up period. Patients reported improvements in their quality of life and reductions in pain and disability. In addition, the consumption of analgesic drugs was reduced.

### 3.4. Complications of O_2_-O_3_ Treatment

No significant complications of O_2_-O_3_ treatment were observed in the mentioned studies. Beyaz and Sayhan [18] reported one patient with hoarseness at 3 days after intradiscal O_2_-O_3_ application, with spontaneous resolution over a period of 1 week. Further, some patients reported some discomfort such as neck stiffness, transient postprocedural increases in pain, dysphagia, and sore throat. All these problems disappeared spontaneously within one day after the procedure [18]. Raeissadat et al. [13] reported minor adverse reactions in two patients (one in the O_2_-O_3_ group and the other in the dry needling group) that occurred within the first day after injection and required no treatment.

## 4. Discussion

In recent decades, O_2_-O_3_ therapy has been used for musculoskeletal pain treatment. It has been applied in the treatment of pain in knee osteoarthritis, subacromial tendinopathy, or in combination with shock wave therapy to treat calcific tendinitis of the shoulder. Other described musculoskeletal applications of O_2_-O_3_ therapy include the treatment of rheumatoid arthritis, tendinopathies, neural entrapment syndrome, lateral epicondylitis, rhizarthrosis, spondylolisthesis, spondylolysis, plantar fasciitis, septic spondylodiscitis, Quervain’s tenosynovitis, fibrosis after the resection of Morton’s neuroma, and pathology of the temporomandibular joint. However, the most therapeutic musculoskeletal applications of O_2_-O_3_ mixture are performed in the spine region [19].

The majority of the literature dealing with the minimally invasive O_2_-O_3_ treatment of spinal pain is focused on the lumbar region. This is probably due to the fact that pain of the lumbar spine is up to 10 times more common in comparison to the cervical counterpart [37]. Commonly used applications of O_2_-O_3_ therapy on lumbar spine encompass intradiscal, periganglionic, periradicular, and paravertebral injections. Meta-analyses dealing with the efficacy of the O_2_-O_3_ treatment of lumbar spine pain usually involve hundreds of patients [26,38,39]. Steppan et al. [26] conducted a meta-analysis of the effectiveness and safety of ozone treatments for lumbar HDs. Based on the study results, authors consider O_2_-O_3_ treatment to be effective and very safe. Moreover, compared to surgical discectomy, the O_2_-O_3_ treatment of HDs is associated with a much lower incidence of complications, and the recovery time is also considerably shorter. Magalhaes et al. [39] conclude that the indicated level of evidence for long-lasting pain reduction is II-3 for intradiscal O_2_-O_3_ application and II-1 for paravertebral O_2_-O_3_ application. Further, the grading of the recommendation is 1C for O_2_-O_3_ therapy applied intradiscally and 1B for O_2_-O_3_ therapy applied paravertebrally [39].

All evaluated studies utilized VAS or NRS scales to measure the changes in pain intensity at the follow-up. The baseline VAS or NRS values were similar in the evaluated studies. The lowest initial mean VAS (6.2 ± 0.9 points) was given in the lidocaine group in the study by Raeissadat et al. [13], and the highest mean VAS (8.5 ± 1.3 points) was observed in the study by Ucar et al. [36]. Significant decreases in VAS or NRS were observed in all evaluated studies regardless of the cause of pain and the site of O_2_-O_3_ therapy. These scales are also commonly used to assess the effect of O_2_-O_3_ therapy in the treatment of lumbar spine pain. The ODI scale and NDI as its modification for NP are routinely used to measure functional status (disability) in spinal pain. Except for the study by Ucar et al., the NDI and ODI were used in all the evaluated studies. Other parameters assessing treatment outcome (DN4, SF-36, SF-12, Japanese Orthopedic Association score, etc.) have been used non-constantly, e.g., ROM has been assessed only in studies dealing with neck MPS [13,34].

There are two more retrospective studies on O_2_-O_3_ therapy which were not included in this literature review. The first of them is the study by Alexandre et al. [40], dealing with the intradiscal injection of O_2_-O_3_ mixture for the treatment of cervical HDs. The authors, among other results, reported a complete abolition of pain in 79.3% of patients and the amelioration of pain in 9.9% of patients. However, these favorable outcomes of O_2_-O_3_ therapy were not expressed in VAS/NRS or NDI/ODI scales. The second study, by Martinelli et al. [41], focused on the clinical efficacy and safety of intramuscular paravertebral applications of an O_2_-O_3_ mixture in patients with cervicobrachial pain. A significant pain reduction (measured by VAS) at follow-up was observed in all the included patients. Unfortunately, only the abstract of this study was available in electronic form.

The options for accessing the intervertebral disc differ for the cervical and lumbar spine. While the dorsal oblique paravertebral approach is usually used in the lumbar region, only the right anterolateral approach seems to be safe for cervical discs as the left anterolateral approach is avoided to prevent esophageal injury [29,42]. The O_2_-O_3_ mixture can also be applied near the dorsal root ganglion. In the lumbar spine, this can be easily performed together with O_2_-O_3_ nucleolysis, or it can be performed independently as a periradicular therapy [43]. Further, the paravertebral application of the O_2_-O_3_ mixture is used on both the lumbar and cervical spine; this treatment can be effective in treating pain even in patients diagnosed with intervertebral HD [36,44]. It is also possible to use the application of O_2_-O_3_ mixture in the treatment of facet joint syndrome [45].

Studies dealing with cervical disc nucleolysis [18,29] utilized the fluoroscopic (plat panel c-arm) guidance. In both studies, the patients were placed in supine position during the needle’s introduction. In the study by Ghatge et al. [29], the needle was introduced through the space between the manually displaced carotid artery and trachea/esophagus. Beyaz and Sayhan [18] used an anterolateral approach with the introduction of the needle through the larynx and jugular-carotid vessels with laryngeal subluxation. Performing intradiscal nucleolysis on the lumbar spine seems to be less complicated as navigation in this area is usually provided by fluoroscopy or CT. In addition, in the case of CT navigation, the operator does not have to be exposed to ionizing radiation [46]. Paravertebral access on the cervical and lumbar spine is usually performed in patients in prone position. In this case, fluoroscopy, CT, or ultrasonography can be used for imaging guidance. The use of ultrasonography for navigation is very advantageous due to the lack of risk of stochastic effects of ionizing radiation. Ultrasonography allows for the location of landmark structures on the cervical spine. Sagittal scanning allows for the precise definition of the intervertebral levels, while transverse scanning shows the medial paravertebral muscles as the site of O_2_-O_3_ injection [32]. Rimeika et al. [47] performed a meta-analysis of the literature on percutaneous O_2_-O_3_ injections, comparing image-guided to non-image-guided techniques for low back pain treatment. The authors stated that procedures utilizing imaging guidance showed better performances, including higher therapeutic efficacy in comparison to the techniques based only on anatomical landmarks.

The effectiveness and possible side effects of O_2_-O_3_ therapy depend on the concentration and amount of O_2_-O_3_ mixture applied. At high doses, the effect of O_2_-O_3_ mixture may be detrimental, and at a too low dose, the beneficial therapeutic effect may not occur (hormetic effect of ozone) [48]. The study by Niu et al. [49] investigated the therapeutic effect of different concentrations of O_2_-O_3_ mixture on post-traumatic lumbar HDs. The authors found that low concentrations of the O_2_-O_3_ mixture (20 μg/mL and 40 μg/mL) lead to a decrease in IL-6, IgG, and IgM expression in serum, resulting in analgesic and anti-inflammatory effects. On the other hand, high concentrations of the O_2_-O_3_ mixture (60 μg/mL) lead to an increase in the expression of IL-6, IgG, and IgM in the serum, resulting in painful and pro-inflammatory effects. The optimal O_2_-O_3_ concentration for the treatment of lumbar HDs was found to be 40 μg/mL. It can be assumed that an intradiscally applied O_2_-O_3_ mixture will have a similar effect on the nucleus pulposus in the lumbar and cervical spine. In this respect, the concentrations used in studies targeting the cervical spine (Ghatge et al.—30 μg/mL, Beyaz and Sayhan—20 μg/mL) can be considered appropriate. However, the difference in the amount of O_2_-O_3_ mixture applied (Ghatge et al.—1–2 mL, Beyaz and Sayhan—4–5 mL) was noticeable [18,29]. Paravertebral intramuscular O_2_-O_3_ therapy is usually performed as a series of injections administered sequentially over several weeks. Therefore, the applied volume of the O_2_-O_3_ mixture is overall larger compared to the solitary intradiscal application.

The concentration of the O_2_-O_3_ mixture for paravertebral application must be neither below 18–20 µg/mL nor higher than 25 µg/mL. Treatment is not effective at too low O_2_-O_3_ concentrations. On the other hand, higher concentrations of the O_2_-O_3_ mixture can cause pain, in particular during the first treatment applications. However, there are observations that after five to seven applications, the pain threshold increases, and therefore the concentration of the O_2_-O_3_ mixture can be slowly increased. The limit of 30 mg/mL should not be exceeded [25,50].

The procedure of cervical intradiscal O_2_-O_3_ nucleolysis may be supported by further medication. Beyaz and Sayhan [18] used fentanyl and midazolam for sedation and lidocaine for cutaneous and subcutaneous local anesthesia. Gentamicin sulfate was administered as antibiotic prophylaxis. Before the needle was inserted to the disc, 16 mg of dexamethasone and 2 mL of 0.5% bupivacaine were injected into the epidural space from C6 to C7 or into the C7-T1 interlaminar space utilizing fluoroscopy guidance. Ghatge et al. [29] mentioned triamcinolone injection in the deltoid muscle on the affected side.

No serious complications were reported in the reviewed studies. The reported minor complications in two studies [13,18] resolved spontaneously, without the need of further treatment. It is uncertain whether these complications were definitely related to the O_2_-O_3_ treatment. Boyce et al. [51] in their research article determined and reported the type of adverse events associated with the utilization of dry needling. Information related to minor and major adverse events that occurred during 20,464 dry needling treatment sessions was collected. The authors concluded that expected minor adverse events such as mild bleeding, bruising, and pain during dry needling were common, and major adverse events were rare. Based on the findings of this study, the overall risk of a major adverse event during dry needling is small. Complications of spinal pain treatment utilizing O_2_-O_3_ therapy are rare; however, unfortunately, they can be serious. Needle introduction at the level of the cervical spine can cause mechanic damage, where vascular structures, nerves, and the structures of the respiratory and digestive tract may be injured. Strict adherence to the rules of asepsis is essential to minimize the risk of infectious complications. There is also the possibility of an adverse reaction due to the inadvertent intravascular application of O_2_-O_3_ mixture. Andrés-Cano et al. [52] presented a case of cervical spondylodiscitis following intradiscal O_2_-O_3_ nucleolysis for HD. During treatment, the Beta-hemolytic streptococcus was isolated. This finding could suggest a transesophageal puncture during disc O_2_-O_3_ therapy. Freund et al. [53] described a case of a patient who developed neurologic symptoms after the paravertebral administration of O_2_-O_3_ mixture. CT revealed the presence of gas in the right vertebral artery, and MRI of the brain depicted multiple infarcts in posterior circulation. Most of the complications reported in the literature are related to O_2_-O_3_ therapy performed in the lumbar spine. Corea et al. [54] presented a case of stroke in posterior circulation during O_2_-O_3_ therapy. Authors consider gas embolization as a probable reason for this adverse event. Ginanneschi et al. [55] presented a case of ventral and dorsal root injury occurring after the transcutaneous intradiscal infiltration of O_2_-O_3_ for L4-L5 HD. The mechanism underlining this injury was not clear; the authors hypothesized a possibility of a transitory increase in cerebrospinal fluid pressure following disc injection. Menéndez et al. [56] described a case of purulent complication following lumbar paravertebral injections of O_2_-O_3_ therapy. Lo Giudice et al. [57] reported a case of vitreous–retinal hemorrhages with bilateral visual loss after the intradiscal and periganglionic injection of O_2_-O_3_ mixture for lumbar HD. Toman et al. [58] presented a case of pneumocephalus, manifested by a rapidly evolving severe headache in patient who underwent percutaneous epidural neuroplasty combined with ozone treatment for FBSS. Vaiano et al. [59] described a case of cortical blindness as a consequence of bilateral occipito-parietal lobe ischemia after the intradiscal and periganglionic O_2_-O_3_ application at the L5/S1 level.

A lack of the standardization of O_2_-O_3_ therapy should be considered a major limitation of this review. There are significant differences in the number of O_2_-O_3_ injections and the volume and concentration of the O_2_-O_3_ mixture used (Table 1). Therefore, the possibility of a comparison between the evaluated studies is limited. The standardization of the protocols should be essential for future studies dealing with O_2_-O_3_ therapy for pain management.

Authors are aware that the time span of the literature search between 01/2000 and 01/2024 can be considered a limitation of this review. However, in an initial search for papers dealing with the O_2_-O_3_ treatment of musculoskeletal NP, we did not find any published before 01/2000. For this reason, the scope of the literature search is limited to the specified time range. Minimally invasive O_2_-O_3_ pain treatment can still be considered a relatively new method. For example, the cited review papers dealing with the O_2_-O_3_ treatment of discogenic lumbar spine pain include the earliest studies from 1998 [39], 2003 [26], and 2005 [38].

Limitations of this review also include the fact that the timing of minimally invasive O_2_-O_3_ treatment was not specified in the two evaluated studies [29,36], although the inclusion of patients with chronic pain may be considered here. The other evaluated studies clearly declare the realization of O_2_-O_3_ treatment in patients with chronic pain. In practice, the minimally invasive pain treatment methods are mostly used in patients with chronic pain when conservative treatment is not sufficient [60].

## 5. Conclusions

Currently, there are only a few clinical trials evaluating the treatment of musculoskeletal NP using O_2_-O_3_ therapy. Moreover, most of these studies are designed as observational. Nevertheless, data from the available literature suggest that minimally invasive O_2_-O_3_ therapy of musculoskeletal NP may be potentially beneficial and relatively safe.

However, there is still a lack of proven benefits of O_2_-O_3_ therapy compared to conservative treatments of musculoskeletal NP. O_2_-O_3_ therapy may be considered for patients who do not benefit from conservative treatment approaches.

The etiology of musculoskeletal NP is quite heterogeneous. From this point of view, it can be expected that the effect of O_2_-O_3_ therapy on other causes of musculoskeletal NP, such as cervical facet joint syndrome, will be investigated in the future.

## Figures and Tables

**Table 1:** A summary of data from the evaluated studies.

Author, Year, Study Type	No. of Subject	Disease Duration	Target	Imaging Guidance	O_2_-O_3_ Vol. Concentration	No. of Injections	Control Groups	Outcome	Follow-up Terms	Post-Treatment Outcome
Korkmaz et al., 2023, RCT [34]	45	MPS chronic	TP	without	5 mL 10 μg/mL	3 × 1 per week	LD	VAS, NDI, ROM, PS	W4, W12	Significant decrease in VAS, PS, and NDI scores in both O_2_-O_3_ and LD groups. LD more effective at reducing pain. Non-significant change in ROM.
Raeissadat et al., 2018, RCT [13]	62	MPS chronic	TP	without	8 mL 15 μg/mL	3 × 1 per week	LD DN	VAS, NDI, ROM, PPT	W4	Significant decrease in VAS and NDI and increase in PPT in all groups. ROM without significant change. O_2_-O_3_ and LD more effective than DN.
Ghatge et al., 2022, PS [29]	246	HD unspecific	ID	fluoro	1–2 mL 30 μg/mL	1	-	VAS, ODI, modified McNab criteria	M1, M3, M6, Y1	Significant decrease in VAS and ODI at all follow-up terms. Modified McNab criteria indicated recovery: excellent in 56.1%, good in 20.3%, and fair in 8.9%.
Beyaz and Sayhan, 2018, PS [18]	44	HD, NP chronic	ID	fluoro	4–5 mL 20 μg/mL	1	-	VAS, ODI	W2, W6, M6	Significant pain relief at all follow-up terms. S uccessful outcomes (2-point decrease in VAS and 15-point decrease in ODI) at a rate of 93.1%, 95.4%, and 97.7% at follow-up terms.
Rania et al., 2022, RS [35]	540	NP, CBP chronic	IM	without	30 mL 10 μg/mL	11.7 ± 3	-	NRS, DN4, SF-36, ADR, ADI	after 8, 12 procedures, Y1	Significant decrease in VAS and DN4. All patients free of pain 1 year after treatment. Significant improvement in quality of life. No serious ADR recorded. ADI was reduced.
Ucar et al., 2020, RS [36]	72	DNP unspecific	IM	without	30 mL 20 μg/mL	6 × 1 per week	-	VAS, JOAs	M2, M6	VAS and JOAs at both follow-up terms with significant improvement.
Latini et al., 2024, RS [32]	25	NP chronic	IM	US	2 × 3–5 mL 15 μg/mL	12 in 12 weeks	-	NRS, NDI, SF-12, ADI	after procedure, M1, M3, M6	Significant improvement in all outcome measures at each assessment. ADI reduction at each assessment.

Abbreviations: RCT, randomized controlled trial; PS, prospective study; RS, retrospective study; No., number; subject., subjects/patients; MPS, neck myofascial pain syndrome; HD, cervical disc hernia; NP, neck pain; CBP, cervicobrachial pain; DNP, discogenic neck pain; unspecific, duration of pain unspecified; fluoro, fluoroscopy; US, ultrasound; TP, neck trigger point; ID, intradiscal; IM, intramuscular; O_2_-O_3_ vol. concentration, oxygen–ozone gas mixture volume and concentration; LD, lidocaine; DN, dry needling; VAS, Visual Analog Scale; NRS, Numerical Rating Scale; ODI, Oswestry Disability Index; NDI, Neck Disability Index; ROM, Range of Motion in lateral cervical flexion; PS, pain score (by applying pressure on trigger point); PPT, pain pressure threshold (defined as the minimum amount of compression on the trigger point that reproduces the pain); DN4, (Neuropathic Pain 4 Questions survey); SF-36, 36-Item Short Form Health Survey; SF-12, 12-Item Short Form Health Survey; JOAs, Japanese Orthopedic Association score; ADR, adverse drug reaction; ADI, analgesic drug intake; W, week; M, month; Y, year.
